# Skeletal muscle mass index as a predictor of long-term cirrhosis onset in young non-cirrhotic males with acute-on-chronic liver failure

**DOI:** 10.3389/fnut.2022.1071373

**Published:** 2022-12-22

**Authors:** Jie Bai, Manman Xu, Fengling Peng, Junwei Gong, Jinqiu Zhao, Xiaodong Song, Yongguo Li

**Affiliations:** ^1^Department of Infectious Diseases, The First Affiliated Hospital of Chongqing Medical University, Chongqing, China; ^2^Fourth Department of Liver Disease (Difficult and Complicated Liver Diseases and Artificial Liver Center), Beijing You’an Hospital Affiliated to Capital Medical University, Beijing, China; ^3^Department of Radiology, The First Affiliated Hospital of Chongqing Medical University, Chongqing, China; ^4^Department of Neurology, Peking University People’s Hospital, Beijing, China

**Keywords:** acute-on-chronic liver failure, skeletal muscle mass index, cirrhosis, prognosis, long-term, male

## Abstract

**Background:**

The relationship between skeletal muscle mass index (SMI) and cirrhosis incidence in patients with non-cirrhotic acute-on-chronic (ACLF) has not been clarified. This study aimed to assess the predictive value of SMI on the incidence of long-term cirrhosis in male non-cirrhotic ACLF patients.

**Materials and methods:**

Male ACLF patients who were free of liver cirrhosis were retrospectively included in this study. Univariate and multivariate logistic analyses were conducted to determine the risk factors for the long-term (1-year) development of cirrhosis. The receiver operating characteristic curves (ROC) were used to assess the ability of SMI levels to predict the incidence of cirrhosis. Restricted triple spline (RCS) described the dose-response relationship between SMI and the risk of cirrhosis. Subgroup analysis was stratified by age (≤ 40 years and > 40 years).

**Results:**

A total of 230 subjects were included in this study, of whom 45.2% (104/230) were diagnosed with cirrhosis within 360 days. Patients who progressed to cirrhosis had a lower SMI [46.1 ± 6.9 versus 49.2 ± 6.5 cm^2^/m^2^, *P* = 0.001] and a higher proportion of sarcopenia (19.2% versus 6.3%, *P* = 0.003). In multivariate logistic regression, SMI remained a protective agent against 360-days progression to cirrhosis in males with ACLF after adjustment (OR 0.950, 95% CI: 0.908–0.994, *P* < 0.05). SMI exerted a non-linear dose-dependent effect on the risk of cirrhosis. The area under the ROC curve (AUC) for the L3-SMI to predict the incidence of cirrhosis in male non-cirrhotic ACLF patients was 0.636 (*P* < 0.001). We observed significant differences in SMI among male ACLF patients in different age groups. Further subgroup analysis by age revealed that lower SMI was associated with the 1-year incidence of cirrhosis in male ACLF patients aged less than 40 years (OR 0.908, 95% CI: 0.842–0.979, *P* < 0.05), whereas SMI did not affect the 1-year risk of cirrhosis in older subjects (age > 40 years).

**Conclusion:**

A higher SMI represents an independent protective factor for developing long-term cirrhosis in male ACLF patients who do not experience cirrhosis, especially in those under 40 years of age.

## Introduction

Acute-on-chronic liver failure (ACLF) involves acute and severe hepatic deterioration based on established chronic liver disease (CLD) or cirrhosis, which usually progresses rapidly and leads to unfavorable outcomes (1). Despite the high short-term mortality rates in patients with ACLF, liver function in surviving patients remains reversible (1, 2). With the resolution of acute injury and over time, some patients may regain normal liver function, while others progress to cirrhosis. Therefore, existing scores such as the APASL ACLF Research Consortium (AARC), Model for End-Stage Liver Disease (MELD), and the MELD-Na score are used for assessing disease severity and guiding organ allocation (1, 3, 4), but not for predicting the occurrence of cirrhosis in survivors. Exploring the occurrence of cirrhosis post-ACLF is beneficial to understand ACLF better and improve prognosis.

Sarcopenia is the severe loss of skeletal muscle mass and function (5). Sarcopenia is strongly associated with adverse clinical outcomes involving falls, decreased function, weakness, and mortality (6). Approximately 70% of patients with the advanced liver disease suffer from sarcopenia (7). Available studies have demonstrated the impact of sarcopenia on the poor prognosis of patients with liver disease (8–12). Skeletal muscle index at the level of the third lumbar vertebra (L3-SMI), based on computer tomography (CT) scans, is a well-described indicator for assessing total skeletal muscle and sarcopenia (13–15). Previous studies have investigated the relationship between L3-SMI and ACLF mortality and suggested that lower L3-SMI may serve as a risk predictor for mortality in ACLF patients (16, 17). Yet, there is a lack of evidence concerning the relationship between L3-SMI and the incidence of long-term cirrhosis in ACLF patients free of cirrhosis.

In the present research, we intended to investigate the predictive value of L3-SMI on the incidence of long-term cirrhosis in male non-cirrhotic ACLF patients. Additionally, we explored the effect of L3-SMI on different age groups (age ≤ 40 years and age > 40 years), as there were significant differences in L3-SMI levels between groups.

## Materials and methods

### Patients

We retrospectively identified inpatient ACLF patients who were admitted to the First Affiliated Hospital of Chongqing Medical University between October 2012 and July 2021. ACLF was diagnosed according to the criteria by Asia-Pacific Association for the Study of the Liver (APASL), which included jaundice [total bilirubin (TB) ≥ 5 mg/dL] and coagulation dysfunction [international normalized ratio (INR) ≥ 1.5], with the onset of ascites and/or hepatic encephalopathy (HE) within 4 weeks (1). Enrolled subjects were over 18 years of age, received abdominal CT examination within 2 weeks after admission, and were followed for a least 360 days. Exclusion criteria were (1) history of cirrhosis; (2) diagnosis of hepatocellular carcinoma (HCC) or other malignancies; (3) suffering from other disorders causing malnutrition such as tuberculosis, hyperthyroidism, or neuromuscular disease; (4) receiving long-term corticosteroid therapy; (5) death or undergoing liver transplant surgery.

This study was approved by the Ethics Committee of the First Affiliated Hospital of Chongqing Medical University (2022-K434). The requirement to obtain informed consent from patients was waived because the study was retrospective in design.

### Clinical data

Demographic information and laboratory findings were collected on admission, including gender, age, height, weight, blood routine, liver and kidney function tests, electrolytes, and coagulation function. We calculated the prognostic scores MELD and MELD-Na for every patient by using the values of the measured parameters of interest.

Diagnosis of cirrhosis was based on clinical, biochemical, radiological (including ultrasound, elastography, CT and MR) and endoscopic findings related to cirrhosis and/or portal hypertension, or on liver biopsy findings (18). For all registered patients, physicians assessed information on cirrhosis at baseline and 360 days after enrollment.

### Evaluation of psoas muscle index

The area of the skeletal muscle (including psoas major, erector spinae, transverse abdominis, internal and external obliques and rectus abdominis) at the L3 level was measured using the 3D Slicer software (version 5.1.0). SMI was calculated by dividing the area of the skeletal muscle at the L3 level by the square of the height (m^2^) (13, 14). Analysis of the abdominal CT scans was performed independently by two imaging physicians. A third physician was involved in reaching a consensus whenever disagreement arose.

### Statistical analysis

Statistical analyses were conducted using IBM SPSS Statistics (version 22.0) and R software (version 4.1.2). Continuous variables were expressed as the mean ± standard deviation or median (interquartile spacing) of a normal or non-normal distribution. Categorical variables were shown as numbers (percentages). Comparisons between the two groups were calculated using unpaired *t*-test for parametric data, Mann-Whitney *U* test for non-parametric data and chi-square test for categorical data. Univariate and multivariate (forward-stepwise method) logistic regression analyses were conducted to examine potential predictors of cirrhosis. Bilateral *P* values < 0.05 considered statistically significant.

The receiver operating characteristic curves (ROC) were used to assess the ability of SMI levels to predict the incidence of cirrhosis. Moreover, we constructed a restricted cubic spline (RCS) describing the relationship between SMI and the risk of cirrhosis, according to the three knots of the SMI level distribution, i.e., the 10th, 50th, and 90th percentiles.

## Results

### Baseline patient characteristics

**FIGURE 1:**
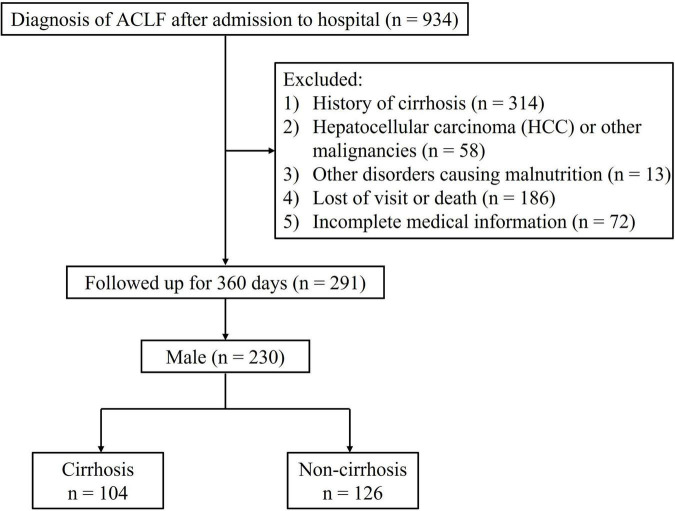
Study flow diagram. ACLF, acute-on-chronic liver failure.

A total of 230 male ACLF patients without cirrhosis at baseline were included in this study (Figure 1). The baseline characteristics of the individuals involved are shown in Table 1. During the 360-days follow-up period, 45.2% (104/230) of patients progressed to cirrhosis. Overall, the cirrhosis group was associated with higher mean age, white blood cell count (WBC), international normalized ratio (INR), total bilirubin (TB), MELD score, and MELD-Na score, but lower albumin and blood sodium levels. Patients who progressed to cirrhosis at 1 year presented with a lower SMI [46.1 ± 6.9 versus 49.2 ± 6.5 cm^2^/m^2^, *P* = 0.001] and a higher proportion of sarcopenia (19.2% vs. 6.3%, *P* = 0.003). In addition, patients in the cirrhosis group suffered a greater incidence of hepatic encephalopathy and ascites at baseline (*P* < 0.05).

**TABLE 1:** The baseline characteristics between cirrhosis and non-cirrhosis groups.

Variable	All patients (*n* = 230)	Non-cirrhosis group (*n* = 126)	Cirrhosis group (*n* = 104)	*P*-value
Age (years), mean (SD)	43.4 (14.1)	40.4 (13.3)	46.9 (14.3)	< 0.001
BMI (kg/m^2^), mean (SD)	23.0 (3.7)	23.1 (3.2)	22.9 (4.3)	0.750
SMI (cm^2^/m^2^), mean (SD)	47.8 (6.8)	49.2 (6.5)	46.1 (6.9)	0.001
Sarcopenia (SMI < 40.2 cm^2^/m^2^), n (%)	28 (12.2)	8 (6.3)	20 (19.2)	0.003
Hepatic encephalopathy, n (%)	34 (14.8)	2 (1.6)	32 (30.8)	< 0.001
Ascites, n (%)	146 (63.5)	72 (57.1)	74 (71.2)	0.028
WBC (× 10^9^/L), median (IQR)	6.1 (4.7–7.9)	5.7 (4.6–7.4)	6.7 (4.9–8.3)	0.008
HB (× 10^9^/L), mean (SD)	134.2 (19.9)	135.5 (17.8)	132.5 (22.1)	0.254
PLT (× 10^9^/L), median (IQR)	130.0 (95.3–164.0)	134.0 (102.8–170.3)	121.5 (91.3–153.5)	0.079
INR, median (IQR)	1.9 (1.7–2.3)	1.8 (1.6–2.1)	2.1 (1.8–2.5)	< 0.001
TB (mg/dL), median (IQR)	13.7 (9.5–19.6)	12.7 (9.0–18.1)	15.1 (10.8–21.1)	0.002
ALB (g/L), mean (SD)	32.7 (5.0)	33.8 (5.3)	31.3 (4.4)	< 0.001
Na (mmol/L), mean (SD)	138.6 (3.6)	139.4 (2.9)	137.6 (4.0)	< 0.001
CR (μmol/L), median (IQR)	67.0 (59.0–75.0)	68.0 (60.0–74.3)	66.0 (56.0–75.8)	0.252
MELD score, mean (SD)	24.5 (4.1)	23.4 (3.5)	25.9 (4.4)	< 0.001
MELD-Na score, mean (SD)	24.9 (4.2)	23.5 (3.5)	26.5 (4.4)	< 0.001
Smoking, n (%)	143 (62.2)	76 (60.3)	67 (64.4)	0.523
Alcohol use, n (%)	57 (24.8)	26 (20.6)	31 (29.8)	0.109

SD, standard deviation; IQR, interquartile range; BMI, body mass index; SMI, skeletal muscle mass index; WBC, white blood cell count; HB, hemoglobin; PLT, platelet; INR, international normalized ratio; TB, total bilirubin; ALB, albumin; Na, serum sodium; CR, serum creatinine; MELD, model for end-stage liver disease.

### SMI and long-term (1-year) incidence of cirrhosis in male non-cirrhotic ACLF patients

**FIGURE 2:**
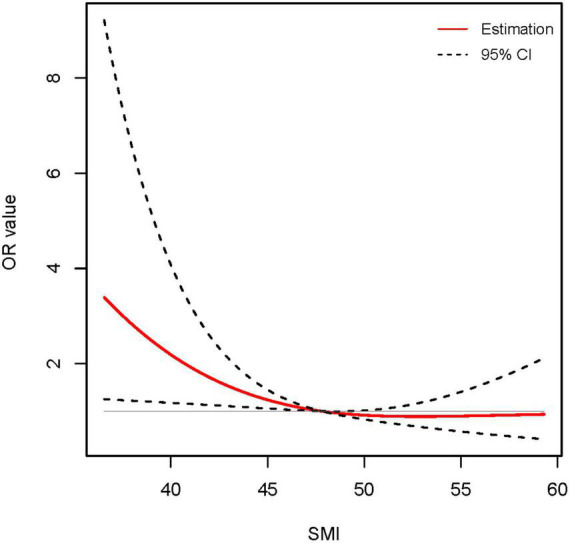
Association between skeletal muscle mass index (SMI) level and long-term (1-year) incidence of cirrhosis using restricted cubic spline (RCS) analysis (*P* for non-linear = 0.127). Results were corrected with age, international normalized ratio (INR), total bilirubin (TB), and serum sodium.

To explore the potential association between SMI and long-term incidence of cirrhosis in male non-cirrhotic ACLF patients, univariate and multivariate logistic regression analyses were performed. Univariate logistic regression analyses revealed that age, WBC, INR, and TB were risk factors for long-term cirrhosis onset, while SMI, albumin and blood sodium served as protective factors (Table 2). Next, the above factors were included in a multivariate regression analysis. We found that after adjusting for other factors, high levels of SMI remained a protective agent for 360-days progression to cirrhosis in men with ACLF (OR 0.950, 95% CI: 0.908–0.994, *P* < 0.05) (Table 2 and Supplementary Figure 1). The RCS analysis demonstrated an approximately linear dose-dependent effect of SMI on the risk of cirrhosis (*P* for non-linear = 0.127) (Figure 2). The area under the ROC curve (AUC) for the L3-SMI to predict the incidence of cirrhosis in male non-cirrhotic ACLF patients was 0.636 (*P* < 0.001) (Supplementary Figure 2).

**TABLE 2:** Univariate and multivariate logistic regression analysis of cirrhosis.

Variable	Univariate		Multivariate	
	**OR (95% CI)**	***P*-value**	**OR (95% CI)**	***P*-value**
Age (years)	1.035 (1.015–1.055)	0.001	1.032 (1.010–1.055)	0.004
SMI (cm^2^/m^2^)	0.932 (0.894–0.971)	0.001	0.950 (0.908–0.994)	0.028
WBC (× 10^9^/L)	1.116 (1.020–1.222)	0.017		
INR	2.283 (1.415–3.682)	0.001	2.324 (1.329–4.063)	0.003
TB (mg/dL)	1.065 (1.024–1.107)	0.001	1.058 (1.014–1.104)	0.010
ALB (g/L)	0.901 (0.850–0.954)	< 0.001		
Na (mmol/L)	0.861 (0.794–0.934)	< 0.001	0.894 (0.818–0.978)	0.014

SMI, skeletal muscle mass index; WBC, white blood cell count; INR, international normalized ratio; TB, total bilirubin; ALB, albumin; Na, serum sodium; OR, odds ratio; CI, confidence interval.

### SMI in male ACLF patients of different age subgroups

**FIGURE 3:**
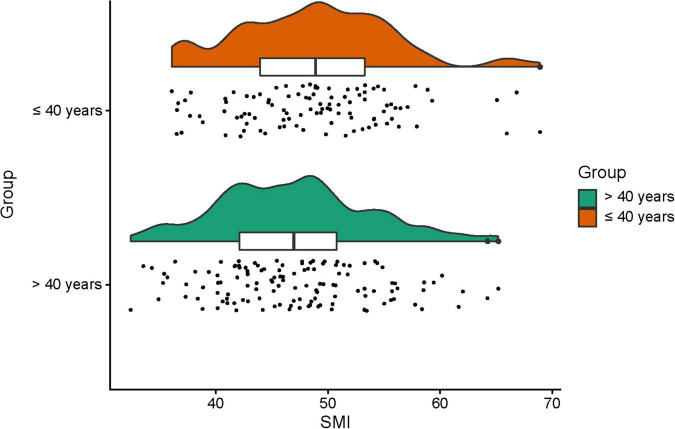
The skeletal muscle mass index (SMI) in different age subgroups (age ≤ 40 years and age > 40 years) in male acute-on-chronic liver failure (ACLF) patients.

It has been established that skeletal muscle mass is gradually reduced over age (19). Our previous study revealed that muscle loss is particularly pronounced after the age of 40 (13). Besides, we found that L3-PMI was independently associated with 1-year mortality in male ACLF patients younger than 40 years, which was not observed in male ACLF patients beyond 40 years (20). Thus, we assessed SMI levels in the age ≤ 40 years and > 40 years groups, respectively. The mean values of SMI for the older and younger groups were 47.0 ± 6.8 and 48.8 ± 6.8 cm^2^/m^2^, respectively (*P* < 0.05). The distribution of SMI in the two groups was shown in Figure 3, exhibiting statistical differences (*P* < 0.05).

### SMI and long-term (1-year) incidence of cirrhosis in male ACLF patients younger than 40 years

Since there were significant differences in SMI among males with ACLF in different age groups, we further explored the connection between SMI and long-term progression to cirrhosis in various age groups. Of 103 male subjects younger than 40 years, 32 (31.1%) suffered liver cirrhosis after 1 year. In this group, the mean SMI of patients who developed cirrhosis at 1 year was 45.8 ± 6.3 cm^2^/m^2^, significantly lower than that of non-cirrhotic patients (50.2 ± 6.6 cm^2^/m^2^, *P* = 0.003).

**FIGURE 4:**
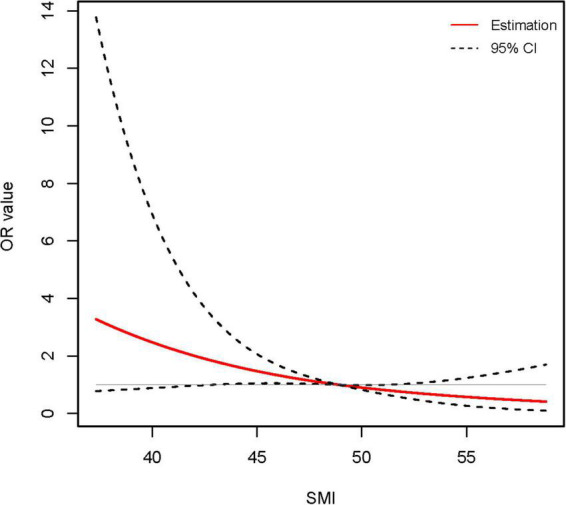
Association between skeletal muscle mass index (SMI) level and long-term (1-year) incidence of cirrhosis in young (age ≤ 40 years) male acute-on-chronic liver failure (ACLF) patients using restricted cubic spline (RCS) analysis (*P* for non-linear = 0.905). Results were corrected with international normalized ratio (INR).

We conducted univariate and multivariate logistic regression analyses of patients’ clinical characteristics. The results indicated that higher INR and TB as well as lower SMI were significantly associated with the risk of 360-days cirrhosis in young men no more than 40 years old with ACLF (Table 3). Multivariate logistic regression identified SMI as a protective factor for the long-term progress to cirrhosis (OR 0.908, 95% CI: 0.842–0.979, *P* < 0.05) (Table 3 and Supplementary Figure 3). Further RCS models revealed an approximately linear dose-response relationship between SMI and the risk of cirrhosis (*P* for non-linear = 0.905) (Figure 4). The area under the ROC curve (AUC) for the L3-SMI to predict the incidence of cirrhosis in young men (age ≤ 40 years) with ACLF was 0.673 (*P* < 0.01) (Supplementary Figure 4).

**TABLE 3:** Univariate and multivariate regression models in different age subgroups in male acute-on-chronic liver failure (ACLF) patients.

Variable	Univariate		Multivariate	
	**OR (95% CI)**	***P*-value**	**OR (95% CI)**	***P*-value**
**age ≤ 40 years**				
Age (years)	0.988 (0.921–1.060)	0.735		
SMI (cm^2^/m^2^)	0.898 (0.835–0.966)	0.004	0.908 (0.842–0.979)	0.012
WBC (× 10^9^/L)	1.176 (1.000–1.383)	0.050		
INR	3.044 (1.451–6.388)	0.003	2.835 (1.286–6.247)	0.010
TB (mg/dL)	1.075 (1.014–1.139)	0.015		
ALB (g/L)	0.918 (0.837–1.006)	0.068		
Na (mmol/L)	0.887 (0.777–1.014)	0.079		
**age > 40 years**				
Age (years)	1.017 (0.981–1.054)	0.362		
SMI (cm^2^/m^2^)	0.962 (0.912–1.014)	0.151		
WBC (× 10^9^/L)	1.071 (0.960–1.195)	0.221		
INR	2.201 (1.096–4.421)	0.027	2.147 (1.066–4.322)	0.032
TB (mg/dL)	1.048 (0.994–1.105)	0.081		
ALB (g/L)	0.921 (0.853–0.995)	0.036		
Na (mmol/L)	0.870 (0.781–0.968)	0.011	0.873 (0.782–0.976)	0.017

SMI, skeletal muscle mass index; WBC, white blood cell count; INR, international normalized ratio; TB, total bilirubin; ALB, albumin; Na, serum sodium; OR, odds ratio; CI, confidence interval.

### SMI and long-term (1-year) incidence of cirrhosis in male ACLF patients older than 40 years

The proportion of male ACLF patients over 40 who developed cirrhosis after 1 year was 56.7% (72/127). The mean SMI of patients with and without cirrhosis after 1 year were 48.0 ± 6.1 cm^2^/m^2^ and 46.3 ± 7.1 cm^2^/m^2^, respectively, not showing a statistical difference (*P* = 0.150). There was no association between SMI and the occurrence of 360-days cirrhosis in older (age > 40 years) male ACLF patients in both univariate and multivariate regression analyses (Table 3). The results of multivariate regression analysis declared that INR and serum sodium were independent predictive factors for the development of 360-days cirrhosis in older men with ACLF (Supplementary Figure 5).

## Discussion

This study firstly investigated the association of L3-SMI with long-term (1-year) cirrhosis progression in male ACLF patients who did not experience cirrhosis. We found that a higher SMI could act as an independent protective factor for progression to cirrhosis, possibly in a linear dose-dependent manner. Subgroup analysis according to different ages indicated that SMI could predict the 1-year risk of cirrhosis for younger (age ≤ 40 years) male ACLF patients, but displayed weak predictive value in older (age > 40 years) individuals.

Acute-on-chronic liver failure (ACLF) is a rapidly progressive hepatic decompensation in the context of CLD, characterized by high short-term mortality and adverse clinical outcome (21–23). Early prediction and improvement of the prognosis of ACLF present a great challenge to clinicians. Almost all existing studies have focused on the survival rate of ACLF. Indeed, liver reserve in ACLF is reversible (1). In patients with no underlying cirrhosis, some of them recover completely, while others progressively develop cirrhosis with impaired quality and longevity. Sarcopenia contributes to unfavorable outcomes in patients with liver disease, of which ACLF is no exception (9, 12, 16, 24, 25). Previous studies have revealed that a high PMI or SMI reduces the risk of death in patients with ACLF (12, 16, 20). Our findings confirmed the predictive value of SMI in long-term progression to cirrhosis for male ACLF patients in the absence of a history of cirrhosis. The results were stable after adjusting for other important variables that included age, INR, TB, and blood sodium.

Mechanistically, sarcopenia potentially correlates with ACLF and cirrhosis. For one thing, an essential hallmark of ACLF is overactive systemic inflammation accompanied by a massive release of proinflammatory molecules (26), which distort the protein synthesis/breakdown balance and thus lead to muscle depletion. In cirrhotic patients, disorders of bioenergy metabolism, including accelerated starvation and increased gluconeogenesis, can also promote muscle wasting (8). Further, hyperammonemia secondary to severe liver dysfunction results in muscle atrophy through the upregulation of myostatin (27). Accordingly, skeletal muscle secretes various cytokines and proteins, which involve in its crosstalk with the liver (28). Myocytokines, including myostatin, irisin and vitamin D, may facilitate the progression of liver disease (29, 30). It has been reported that myostatin induces liver fibrosis by activating the JNK signaling pathway to activate hepatic stellate cells (HSC) (27). In a word, profound liver damage (ACLF and cirrhosis) leads to sarcopenia; conversely, sarcopenia exacerbates the procession of liver disease. Therefore, it is not surprising that low SMI predicts poor outcomes in ACLF, including the development of cirrhosis. Increasing muscle mass has been shown to improve the prognosis of patients with cirrhosis (31, 32). We hypothesized that reversing muscle mass could also improve the outcome of ACLF. Notably, nutritional supplementation alone is often ineffective; new molecular targeting strategies such as myostatin opponents, mTORC1 activators and mitochondrial protectors are promising therapeutic options (8).

Interestingly, the results of this study detected the predictive value of SMI for the clinical outcome of ACLF in young men up to 40 in age, but not in older men. Similar to this work, our earlier study failed to observe the correlation between PMI and mortality in men older than 40 years with ACLF (20). The possible reason is that, in older individuals, aging has a greater effect on reducing skeletal muscle mass than liver failure.

The present study has some limitations. First, as a retrospective study, there was a statistical retrospective bias. Second, our study was single-center and included a limited number of subjects. Prospective multicenter large-sample studies are needed to validate our findings. Third, SMI is used to assess muscle mass, but not for muscle strength. In the future, we will perform a more comprehensive evaluation of muscle mass and strength in the ACLF population.

In conclusion, lower SMI was independently related to the development of long-term cirrhosis in males with ACLF free of cirrhosis, especially in those no older than 40 years. Further exploration of the impact of targeted sarcopenia interventions on the prognosis of ACLF is warranted.

## Data availability statement

The raw data supporting the conclusions of this article will be made available by the authors, without undue reservation.

## Ethics statement

The studies involving human participants were reviewed and approved by Ethics Committee of the First Affiliated Hospital of Chongqing Medical University. Written informed consent for participation was not required for this study in accordance with the national legislation and the institutional requirements.

## Author contributions

JB and YL: conception and design. JB: patient recruitment. JB, JG, and FP: data collection. JB, MX, and XS: data analysis. JB and XS: writing the draft. JB, MX, JZ, XS, and YL: revising and polishing the manuscript. All authors read and approved the final manuscript.

## References

[1] Sarin S Choudhury A Sharma M Maiwall R Al Mahtab M Rahman S Acute-on-chronic liver failure: consensus recommendations of the Asian pacific association for the study of the liver (APASL): an update. *Hepatol Int.* (2019) 13:353–90. 31172417 10.1007/s12072-019-09946-3 PMC6728300

[2] Sarin S Kumar A Almeida J Chawla Y Fan S Garg H Acute-on-chronic liver failure: consensus recommendations of the Asian pacific association for the study of the liver (APASL). *Hepatol Int.* (2009) 3:269–82. 10.1007/s12072-008-9106-x 19669378 PMC2712314

[3] Moylan C Brady C Johnson J Smith A Tuttle-Newhall J Muir A. Disparities in liver transplantation before and after introduction of the MELD score. *JAMA.* (2008) 300:2371–8. 10.1001/jama.2008.720 19033587 PMC3640479

[4] Jalan R Arroyo V. Organ allocation for patients with acute-on-chronic liver failure: time to look beyond MELD-sodium? *J Hepatol.* (2020) 73:1316–8. 10.1016/j.jhep.2020.06.030 32703585

[5] Cruz-Jentoft A Sayer A. Sarcopenia. *Lancet.* (2019) 393:2636–46. 10.1016/S0140-6736(19)31138-9 31171417

[6] Cruz-Jentoft A Bahat G Bauer J Boirie Y Bruyère O Cederholm T Sarcopenia: revised European consensus on definition and diagnosis. *Age Ageing.* (2019) 48:16–31. 10.1093/ageing/afy169 30312372 PMC6322506

[7] Ponziani F Gasbarrini A. Sarcopenia in patients with advanced liver disease. *Curr Protein Pept Sci.* (2018) 19:681–91. 10.2174/1389203718666170428121647 28462693

[8] Dasarathy S Merli M. Sarcopenia from mechanism to diagnosis and treatment in liver disease. *J Hepatol.* (2016) 65:1232–44. 10.1016/j.jhep.2016.07.040 27515775 PMC5116259

[9] Tantai X Liu Y Yeo Y Praktiknjo M Mauro E Hamaguchi Y Effect of sarcopenia on survival in patients with cirrhosis: a meta-analysis. *J Hepatol.* (2022) 76:588–99. 10.1016/j.jhep.2021.11.006 34785325

[10] Tandon P Montano-Loza A Lai J Dasarathy S Merli M. Sarcopenia and frailty in decompensated cirrhosis. *J Hepatol.* (2021) 75(Suppl 1):S147–62. 10.1016/j.jhep.2021.01.025 34039486 PMC9125684

[11] Petermann-Rocha F Gray S Forrest E Welsh P Sattar N Celis-Morales C Associations of muscle mass and grip strength with severe NAFLD: a prospective study of 333,295 UK Biobank participants. *J Hepatol.* (2022) 76:1021–9. 10.1016/j.jhep.2022.01.010 35085594

[12] Artru F le Goffic C Pageaux G Saliba F Louvet A. Sarcopenia should be evaluated in patients with acute-on-chronic liver failure and candidates for liver transplantation. *J Hepatol.* (2022) 76:983–5. 10.1016/j.jhep.2021.09.004 34536432

[13] Kong M Geng N Zhou Y Lin N Song W Xu M Defining reference values for low skeletal muscle index at the L3 vertebra level based on computed tomography in healthy adults: a multicentre study. *Clin Nutr.* (2022) 41:396–404. 10.1016/j.clnu.2021.12.003 34999334

[14] Bahat G Turkmen B Aliyev S Catikkas N Bakir B Karan M. Cut-off values of skeletal muscle index and psoas muscle index at L3 vertebra level by computerized tomography to assess low muscle mass. *Clin Nutr.* (2021) 40:4360–5. 10.1016/j.clnu.2021.01.010 33516603

[15] Albano D Messina C Vitale J Sconfienza L. Imaging of sarcopenia: old evidence and new insights. *Eur Radiol.* (2020) 30:2199–208. 10.1007/s00330-019-06573-2 31834509

[16] Peng H Zhang Q Luo L Lei S Xiong T Long L A prognostic model of acute-on-chronic liver failure based on sarcopenia. *Hepatol Int.* (2022) 16:964–72. 10.1007/s12072-022-10363-2 35771410 PMC9349113

[17] Li T Xu M Kong M Song W Duan Z Chen Y. Use of skeletal muscle index as a predictor of short-term mortality in patients with acute-on-chronic liver failure. *Sci Rep.* (2021) 11:12593. 10.1038/s41598-021-92087-1 34131260 PMC8206330

[18] Ginès P Krag A Abraldes J Solà E Fabrellas N Kamath P. Liver cirrhosis. *Lancet.* (2021) 398:1359–76. 10.1016/S0140-6736(21)01374-X 34543610

[19] Hamaguchi Y Kaido T Okumura S Kobayashi A Hammad A Tamai Y Proposal for new diagnostic criteria for low skeletal muscle mass based on computed tomography imaging in Asian adults. *Nutrition.* (2016) 32:1200–5. 10.1016/j.nut.2016.04.003 27292773

[20] Xu M Li T Kong M Geng N Song W Guo G Psoas muscle index can be used to predict long-term mortality in young male patients with acute-on-chronic liver failure. *Front Nutr.* (2022) 9:811826. 10.3389/fnut.2022.811826 35252298 PMC8894235

[21] Bajaj J O’Leary J Lai J Wong F Long M Wong R Acute-on-Chronic liver failure clinical guidelines. *Am J Gastroenterol.* (2022) 117:225–52. 10.14309/ajg.0000000000001595 35006099

[22] Arroyo V Moreau R Kamath P Jalan R Ginès P Nevens F Acute-on-chronic liver failure in cirrhosis. *Nat Rev Dis Primers.* (2016) 2:16041. 10.1038/nrdp.2016.41 27277335

[23] Moreau R Gao B Papp M Bañares R Kamath P. Acute-on-chronic liver failure: A distinct clinical syndrome. *J Hepatol.* (2021) 75(Suppl 1):S27–35. 10.1016/j.jhep.2020.11.047 34039489

[24] Yang J Chen K Zheng C Chen K Lin J Meng Q Impact of sarcopenia on outcomes of patients undergoing liver resection for hepatocellular carcinoma. *J Cachexia Sarcopenia Muscle.* (2022) 13:2383–92. 10.1002/jcsm.13040 35854105 PMC9530540

[25] Kim S Kim D. Sarcopenia as a prognostic indicator of liver cirrhosis. *J Cachexia Sarcopenia Muscle.* (2022) 13:8–10. 10.1002/jcsm.12869 34812591 PMC8818618

[26] Hernaez R Solà E Moreau R Ginès P. Acute-on-chronic liver failure: an update. *Gut.* (2017) 66:541–53. 10.1136/gutjnl-2016-312670 28053053 PMC5534763

[27] Jindal A Jagdish R. Sarcopenia: Ammonia metabolism and hepatic encephalopathy. *Clin Mol Hepatol.* (2019) 25:270–9. 10.3350/cmh.2019.0015 31006226 PMC6759436

[28] Bhanji R Narayanan P Allen A Malhi H Watt K. Sarcopenia in hiding: The risk and consequence of underestimating muscle dysfunction in nonalcoholic steatohepatitis. *Hepatology.* (2017) 66:2055–65. 10.1002/hep.29420 28777879

[29] Delogu W Caligiuri A Provenzano A Rosso C Bugianesi E Coratti A Myostatin regulates the fibrogenic phenotype of hepatic stellate cells via c-jun N-terminal kinase activation. *Digest Liver Dis.* (2019) 51:1400–8. 10.1016/j.dld.2019.03.002 31005555

[30] Chakravarthy M Siddiqui M Forsgren M Sanyal A. Harnessing Muscle-Liver Crosstalk to Treat Nonalcoholic Steatohepatitis. *Front Endocrinol.* (2020) 11:592373. 10.3389/fendo.2020.592373 33424768 PMC7786290

[31] Tsien C Garber A Narayanan A Shah S Barnes D Eghtesad B Post-liver transplantation sarcopenia in cirrhosis: a prospective evaluation. *J Gastroenterol Hepatol.* (2014) 29:1250–7. 10.1111/jgh.12524 24443785 PMC4024321

[32] Tsien C Shah S McCullough A Dasarathy S. Reversal of sarcopenia predicts survival after a transjugular intrahepatic portosystemic stent. *Eur J Gastroenterol Hepatol.* (2013) 25:85–93. 10.1097/MEG.0b013e328359a759 23011041

