# Supplementary material for: Skeletal muscle mass index as a predictor of long-term cirrhosis onset in young non-cirrhotic males with acute-on-chronic liver failure
Source: Front Nutr. 2022 Dec 22;9:1071373. doi: 10.3389/fnut.2022.1071373 (PMC9815435; doi:10.3389/fnut.2022.1071373)
Supplement: Supplementary file 1 [file Table_1.DOCX]

**Supplementary Figure 1. Forest plot of odds ratios (OR) for long-term (1-year) incidence of cirrhosis.**


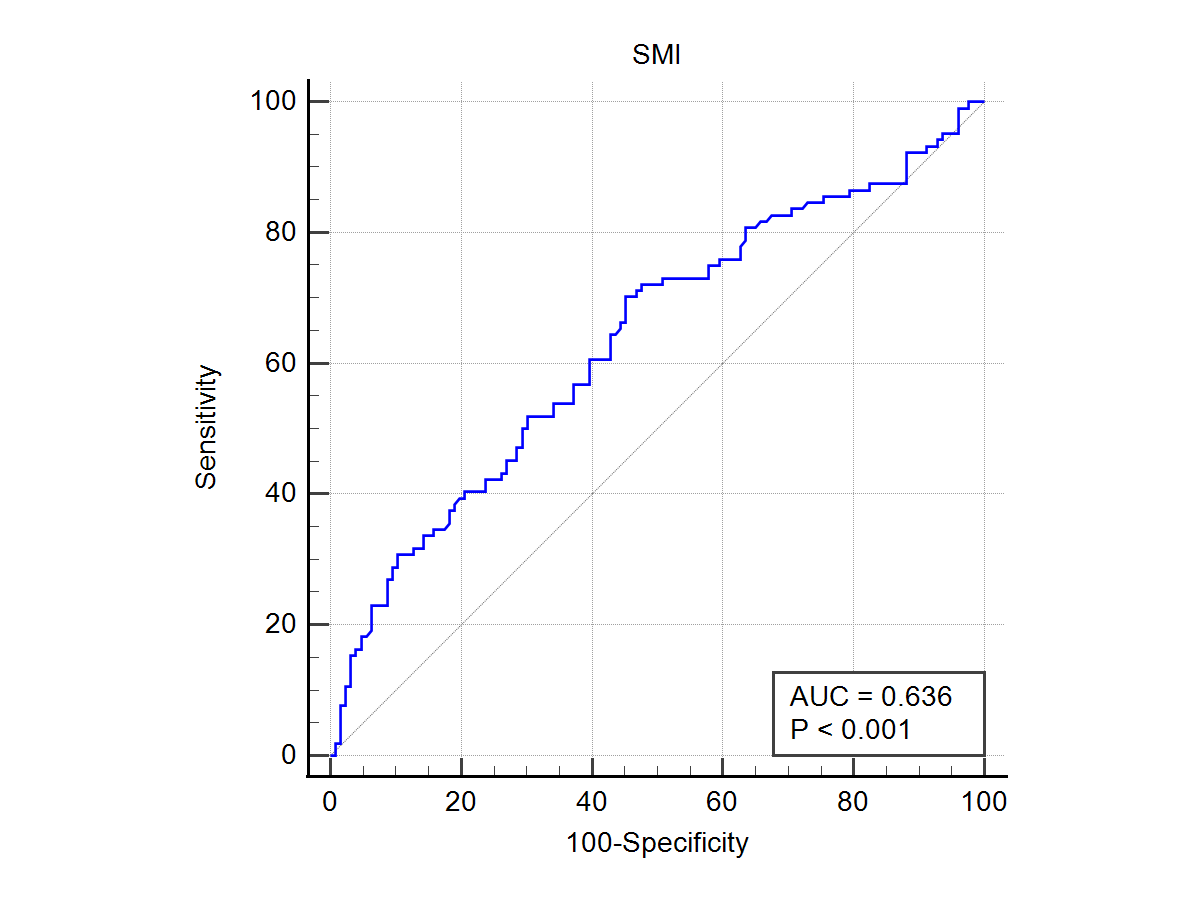


**Supplementary Figure 2. The ROC curve of L3-SMI in predicting the incidence of cirrhosis in male ACLF patients.**





**Supplementary Figure 3. Forest plot of odds ratios (OR) for long-term (1-year) incidence of cirrhosis in young men (age ≤ 40 years) with ACLF**


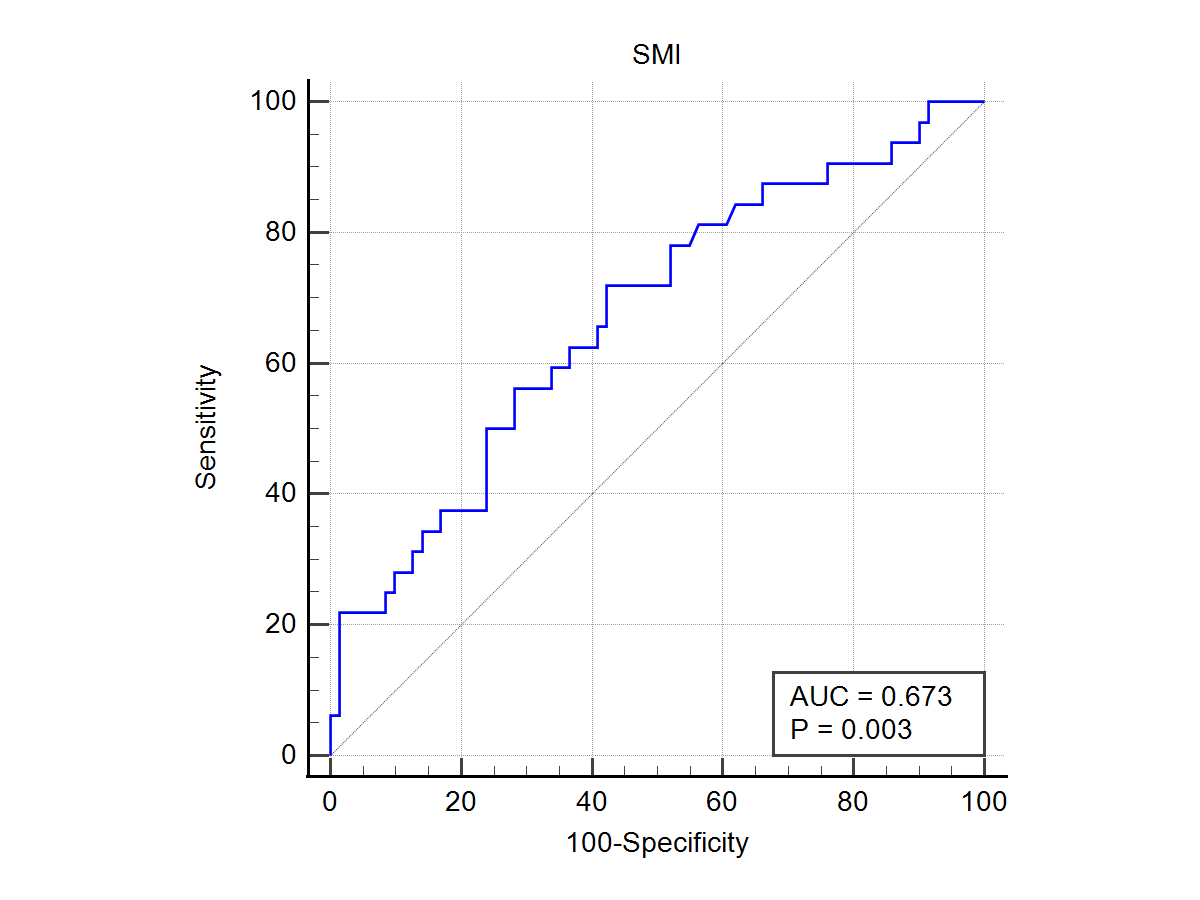


**Supplementary Figure 4. The ROC curve of L3-SMI in predicting the incidence of cirrhosis in young (age ≤ 40 years) male ACLF patients.**





**Supplementary Figure 5. Forest plot of odds ratios (OR) for long-term (1-year) incidence of cirrhosis in old men (age > 40 years) with ACLF**
